# Boron Intake and decreased risk of mortality in kidney transplant recipients

**DOI:** 10.1007/s00394-021-02702-0

**Published:** 2021-10-22

**Authors:** Daan Kremer, Adrian Post, Ulrike Seidel, Patricia Huebbe, Yvonne van der Veen, Dion Groothof, António W. Gomes-Neto, Tim J. Knobbe, Kai Lüersen, Michele F. Eisenga, Gerjan J. Navis, Gerald Rimbach, Stephan J. L. Bakker, TransplantLines Investigators, D. Kremer, T. J. Knobbe, J. H. Annema-de Jong, S. P. Berger, J. Blokzijl, F. A. J. A. Bodewes, M. T. de Boer, K. Damman, M. H. De Borst, A. Diepstra, G. Dijkstra, R. M. Douwes, M. F. Eisenga, M. E. Erasmus, C. T. Gan, A. W. Gomes Neto, H. Grootjans, E. Hak, M. R. Heiner-Fokkema, B. G. Hepkema, F. Klont, H. G. D. Leuvenink, W. S. Lexmond, V. E. de Meijer, H. G. M. Niesters, L. J. van Pelt, R. A. Pol, R. J. Porte, A. V. Ranchor, J. S. F. Sanders, J. C. Schutten, M. J. Siebelink, R. H. J. A. Slart, J. C. Swarte, W. Timens, D. J. Touw, M. C. van den Heuvel, C. van Leer-Buter, M. van Londen, E. A. M. Verschuuren, M. J. Vos, R. K. Weersma, S. J. L. Bakker

**Affiliations:** 1 Department of Internal Medicine, Division of Nephrology, University Medical Center Groningen and University of Groningen, Post Box 30.001, 9700 RB Groningen, The Netherlands; 2 Institute of Human Nutrition and Food Science, University of Kiel, Kiel, Germany; 3 UMC Groningen Transplant Center, University Medical Center Groningen and University of Groningen, Post Box 30.001, 9700 RB Groningen, The Netherlands

**Keywords:** Blue Zones, Mediterranean diet, Trace elements, Wine intake, Transplantation, Inflammation

## Abstract

**Purpose:**

In a search for potentially modifiable factors to improve long-term outcome among kidney transplant recipients (KTR), we hypothesized that boron exposure is associated with improved long-term outcome in KTR.

**Methods:**

We determined 24 h urinary boron excretion using inductively coupled plasma mass spectrometry as a measure of boron exposure in 693 stable KTR (57% male, mean age 53y), enrolled in the TransplantLines Food and Nutrition Biobank and Cohort Study. Dietary intake was assessed using validated food-frequency questionnaires.

**Results:**

Linear regression analyses showed that dietary intake of fruit, wine and nuts were key determinants of boron excretion. In addition, boron excretion was negatively correlated with homocysteine and inflammatory parameters. In total, 73 (32%), 47 (20%) and 30 (13%) KTR died among the lowest, middle and highest tertiles of 24 h urinary boron excretion, respectively (*P*_log-rank_ < 0.001). Cox regression analyses showed that high boron excretion was strongly associated with lower risk of mortality, independent of age, sex, estimated glomerular filtration rate and history of cardiovascular disease (HR per doubling: 0.51, 95% CI: 0.40 to 0.66, *P* < 0.001).

**Conclusion:**

Boron may be an overlooked target to improve long-term survival among KTR and potentially other patients, likely through pathways other than inflammation or the methionine-homocysteine cycle that were previously suggested. Interventional trials are warranted to confirm the potential of dietary boron supplementation in KTR and other patient populations.

**Supplementary Information:**

The online version contains supplementary material available at 10.1007/s00394-021-02702-0.

## Introduction

Even after successful transplantation, kidney transplant recipients (KTR) remain a vulnerable patient group at increased risk of premature mortality [1]. The high mortality risk in KTR is associated with several well-established risk factors, such as hypertension and diabetes, but despite the fact that proper management of these risk factors is in the current treatment guidelines, long-term prognosis has hardly improved over the past decades [1]. This warrants a search for additional, hitherto unknown factors that may be associated with mortality in this vulnerable patient group. In this search, we studied dietary factors in the Blue Zones: regions of the world where a higher than a usual number of people live for more than 100 years, and have a low prevalence of chronic diseases. These regions include Sardinia (Italy), the islands of Okinawa (Japan), Loma Linda (California), Nicoya Peninsula (Costa Rica) and Ikaria (Greece) [2]. We set out to find common factors that may explain the exceptional longevity and physical and cognitive health of the elderly in these zones. One of these factors may be boron exposure.

Boron is a metalloid trace element with atomic number 5, that occurs naturally in the form of boric acid, borax, and several other boron hydrates [3]. Notably, all Blue Zones are either near great boron reserves or near boron-rich oceans [3, 4], and the dietary patterns that are common in the Blue Zones are also high in boron, as they generally include generous amounts of fruits, nuts, vegetables and legumes [2, 5, 6]. Additionally, all Blue Zones are known for high intake of specific boron-rich foods, such as soy products (Loma Linda, Okinawa), beans (Nicoya, Ikaria), and wine (Sardinia) [2, 5, 6]. Given the long average lifespan and the low rates of cardiovascular disease in the boron-rich Blue Zones, we hypothesized that relatively high boron exposure may be associated with low rates of long-term mortality among KTR.

Boron-containing substances have been used by humans for millennia, mainly because of their antiseptic and preservative properties. For example, the Egyptians used boron as a mummification agent, and sodium borate and boric acid were widely used as a food preservative in the past centuries [3]. Potential physiological functions of boron in animals were first recognized in the 1980s, when studies indicated the necessity of boron in bone health and reproduction in zebrafish, chicks, mice, and rats [7–11]. More recent animal and human studies suggest potential beneficial effects of boron in numerous processes throughout the body [12–14]. Our hypothesis that boron exposure may improve patient survival is further supported by a study showing that supplementation of low amounts of boron increased life span in *Drosophila* (i.e. fruit fly) [15]. Additionally, human studies have suggested that boron beneficially affects the cardiovascular system, bone and brain health, and that boron exposure may be associated with a lower risk of cancer and premature mortality [14, 16–18]. Although exact physiological functions remain unclear, boron may exert beneficial effects as a result of anti-inflammatory properties or through the promotion of flux through the homocysteine-methionine cycle [16, 19–21]. In light of these considerations, it may be interesting to not only investigate associations of 24 h urinary boron excretion with dietary intake, but also with clinical and biochemical parameters, including blood pressure, kidney function, circulating lipid concentrations, homocysteine and circulating concentrations of vitamins involved in the homocysteine-methionine cycle.

In this study, we, therefore, measured 24 h urinary boron excretion as a measure of boron exposure among KTR [22, 23]. We aimed to assess associations of clinical, dietary, and biochemical parameters with boron excretion. Finally, we studied prospective associations of boron excretion with mortality and graft failure among KTR.

## Methods

### Study population and design

We included KTR participating in the TransplantLines Food & Nutrition Biobank and Cohort Study [24]. In brief, this prospective cohort study included 707 adult KTR approximately 1 year or longer after transplantation, with a functioning kidney allograft, who visited the outpatient clinic of the University Medical Center Groningen, Groningen, The Netherlands, between November 2008 and June 2011. All patients provided written informed consent. The study was approved by the University Medical Center Groningen institutional review board (METc 2008/186) and adheres to the Declarations of Helsinki and Istanbul (ClinicalTrials.gov: NCT02811835). In total, 693 study participants (98%) had available 24 h urine samples and were included in the current analyses. No patients were lost to follow-up. A diagram visualizing the study population is presented in Figure S1. The primary endpoint of this study was all-cause mortality, and the secondary endpoint was graft failure, which was defined as re-initiation of dialysis and/or retransplantation.

### Clinical parameters and dietary assessment

All measurements were performed during a morning visit to the outpatient clinic. Demographic, clinical, and transplantation-related characteristics were extracted from patient records. Diabetes was defined using the American Diabetes Association criteria [25]. Medication use and medical history were extracted from patient records, and then verified with all patients. Kidney function was assessed by means of the creatinine and cystatin C-based estimated glomerular filtration rate (eGFR), according to the CKD-EPI equation [26]. Dietary intake was assessed using a 177-item validated semi-quantitative food frequency questionnaire (FFQ) [27]. The questionnaires were self-administered, filled out at home and checked by a researcher. To assess the influence of intake of food groups, intake of fruits, vegetables, potatoes, pasta, rice, legumes, nuts, beer, wine, fish, soy products, meat products, coffee, and total plant protein intake was indexed for total energy intake.

### Biochemical analyses

Blood was drawn on the day of the study visit after a fasting period of 8 to 12 h. Urine was collected by participants starting 24 h prior to the study visit, following strict instructions as previously described in detail [28]. Urinary boron concentrations were determined using inductively coupled plasma mass spectrometry (ICP-MS), as summarized in Supplementary Table 1. Plasma vitamin B6 was determined using a validated HPLC method (Waters Alliance) with fluorescence detection (FP-2020; Jasco Inc.) [29]. Other clinical chemistry assays including hemoglobin, creatinine, inflammatory parameters including high sensitivity C-reactive protein (CRP) and leukocyte count, HDL and LDL cholesterol, triglycerides, urinary protein excretion, glucose, vitamin B12, folic acid, and homocysteine were performed using routine laboratory methods (Roche Diagnostics, Basel, Switzerland).

### Statistical analyses

Baseline data of the total population, and according to tertiles of boron excretion were presented as mean ± standard deviation, median [interquartile range (IQR)] or number (percentage) depending on the distribution. Cross-sectional univariable and adjusted linear regression analyses were performed with binary logarithmically transformed (log_2_) 24 h urinary boron excretion as the dependent variable, and the previously mentioned clinical, biochemical and dietary parameters as the independent variable. Normality of the residuals was evaluated by visual inspection of Q–Q plots, where variables were transformed using a log_2_ or square root transformation if necessary to reach assumptions for linear regression.

In the next section, we performed longitudinal analyses. Kaplan Meier curves and log-rank tests were used to visualize and quantify longitudinal differences in patient survival between tertiles of urinary boron excretion. Longitudinal univariable Cox proportional hazards analyses were used to assess associations of tertiles of urinary boron excretion, and urinary boron excretion on a continuous scale with mortality. To account for potential confounding, we adjusted for known predictors of mortality among KTR and variables associated with urinary boron excretion, including age, sex, eGFR, history of cardiovascular disease, smoking status, diabetes, height, weight, LDL cholesterol, HDL cholesterol, and triglycerides. In addition, we adjusted for potential confounding by adjustment of dietary intake of fruit, nuts, fish, wine, plant protein intake and animal protein intake in multivariable Cox regression analyses to the mentioned model. In sensitivity analyses, we also adjusted for potential downstream effects of boron (i.e. plasma homocysteine, high-sensitivity C-reactive protein (hs-CRP)), and other potential confounders involved in the homocysteine-methionine cycle (*i.e.* vitamin B6, vitamin B12, folic acid) in additional multivariable Cox regression analyses. In further sensitivity analyses, we repeated mentioned Cox regression analyses after exclusion of patients that reported current smoking. The assumption of proportional hazards was not violated in any of the models, as assessed using Schoenfeld tests. Hazard ratios (HR) of 24 h urinary boron excretion were presented per doubling with 95% confidence intervals (95% CI), and visualized to facilitate their interpretation. We assessed potential interactions of age, sex and eGFR with boron excretion in the Cox regression analyses. In secondary analyses, similar Cox proportional hazards regression analyses were performed to assess associations of boron excretion with graft failure.

For all cross-sectional analyses, the original non-imputed dataset was used, where variables with > 10 missing values (1.4%) are reported in the table footnotes. For all prospective analyses, multiple imputations using Fully Conditional Specification was performed using the R package ‘mice’, to account for missing data among variables other than data on urinary boron excretion. All data were analyzed using IBM SPSS software, version 23.0 (SPSS Inc., Chicago, IL, USA) and R version 3.5.1 (Vienna, Austria). For all analyses, P-value < 0.05 was considered statistically significant.

## Results

### Baseline characteristics

Baseline characteristics are presented for the total population, and according to tertiles of 24 h urinary boron excretion in Table 1. We included 693 KTR with a mean ± SD age of 53 ± 13 years. Median time after transplantation was 5.4 [2.0 to 12.0] years, and 57% of the study participants were male. Mean ± SD eGFR at baseline was 45 ± 19 ml/min/1.73 m^2^. Median 24 h urinary boron excretion was 1275 [925 to 1729] µg/24 h. Male prevalence increased across increasing tertiles of boron excretion. Furthermore, we observed higher body weight, height, donor age, hemoglobin, and eGFR with increasing tertiles of boron excretion.

**Table 1:** Population characteristics at baseline in tertiles of 24 h urinary boron excretion

	Total population *N* = 693	Tertile 1 *N* = 231	Tertile 2 *N* = 231	Tertile 3 *N* = 231
Urinary boron excretion (µg/24 h)	1275 [925–1729]	840 [695–925]	1275 [1163–1389]	2048 [1727–2600]
Clinical characteristics				
Male sex, *n* (%)	394 (56.9)	117 (50.6)	135 (58.4)	142 (61.5)
Age, yr	53 ± 13	53 ± 14	51 ± 12	55 ± 12
Primary renal disease, *n* (%)				
Unknown	105 (15.2)	36 (15.6)	28 (12.1)	41 (17.7)
Glomerulonephritis	181 (26.1)	60 (26.0)	61 (26.4)	60 (26.0)
Interstitial nephritis	87 (12.6)	27 (11.7)	37 (16.0)	23 (10.0)
Cystic kidney disease	142 (20.5)	43 (18.6)	41 (17.7)	58 (25.1)
Other congenital/hereditary disease	38 (5.5)	17 (7.4)	15 (6.5)	6 (2.6)
Renal vascular disease	38 (5.5)	12 (5.2)	14 (6.1)	12 (5.2)
Diabetes mellitus	36 (5.2)	14 (6.1)	14 (6.1)	8 (3.5)
Other multisystem diseases	48 (6.9)	13 (5.6)	17 (7.4)	18 (7.8)
Other	18 (2.6)	9 (3.9)	4 (1.7)	5 (2.2)
Height, cm	174 ± 10	171 ± 10	174 ± 9	175 ± 9
Weight, kg	80 ± 16	77 ± 16	81 ± 17	83 ± 16
Body surface area, m^2^	1.94 ± 0.22	1.89 ± 0.30	1.95 ± 0.21	1.99 ± 0.21
Body mass index, kg/m^2^	26.6 ± 4.8	26.4 ± 4.9	26.5 ± 4.9	27.1 ± 4.5
Systolic blood pressure, mmHg	136 ± 18	136 ± 18	136 ± 17	136 ± 18
Diabetes, *n* (%)	162 (23.4)	61 (26.4)	60 (26.0)	41 (17.7)
History of cardiovascular disease, *n* (%)	171 (24.7)	49 (21.2)	65 (28.1)	57 (24.7)
Current smoking, *n* (%)	81 (12.5)	32 (13.9)	23 (11.1)	26 (11.7)
Pre-emptive transplantation, *n* (%)	106 (15.3)	29 (12.6)	40 (17.3)	37 (16.0)
Time after transplantation, y	5.4 [2.0–12.0]	6.5 [2.2–12.3]	5.2 [1.8–13.2]	5.0 [1.9–10.5]
Donor age, y	43 ± 15	42 ± 15	41 ± 15	46 ± 16
Living donor, *n* (%)	234 (33.8)	61 (26.4)	79 (34.2)	94 (40.7)
Laboratory measurements				
Hemoglobin, g/dL	13.3 ± 1.7	12.9 ± 1.8	13.4 ± 1.6	13.5 ± 1.8
Creatinine, mg/dL	1.4 [1.1–1.8]	1.6 [1.1–2.0]	1.4 [1.1–1.8]	1.4 [1.1–1.7]
Cystatin C, µg/dL	1.7 [1.3–2.2]	1.9 [1.4–2.6]	1.6 [1.3–2.2]	1.6 [1.3–2.1]
eGFR, mL/min/1.73m^2^	45 ± 19	42 ± 20	47 ± 18	47 ± 17
Leukocyte count, 10^9^/L	8.1 ± 2.6	8.5 ± 3.0	8.2 ± 2.4	7.7 ± 2.3
HDL cholesterol, mmol/L	1.4 ± 0.5	1.3 ± 0.4	1.4 ± 0.5	1.5 ± 0.5
LDL cholesterol, mmol/L	3.0 ± 0.9	3.0 ± 1.0	3.0 ± 1.0	3.0 ± 0.9
Triglycerides mmol/L	1.9 ± 1.0	2.0 ± 0.9	1.9 ± 1.1	1.8 ± 1.0
hs-CRP, µg/dL	1.6 [0.7–4.6]	1.8 [0.8–5.4]	1.6 [0.6–4.8]	1.4 [0.6–3.7]
Urinary protein excretion, g/24 h	0.2 [0.0–0.4]	0.2 [0.0–0.5]	0.2 [0.0–0.4]	0.2 [0.0–0.3]
Homocysteine, µmol/dL	20 [16–25]	22 [17–27]	20 [15–25]	19 [16–23]
Vitamin B12, pmol/L	288 [222–377]	296 [229–370]	285 [212–371]	284 [219–388]
Vitamin B6, nmol/L	29 [17–50]	25 [14–43]	29 [18–49]	33 [21–59]
Folic acid, nmol/L	18 [14–26]	17 [13–23]	18 [13–25]	20 [15–29]
Dietary intake				
Vegetables, grams/day	91 [54 – 123]	91 [48 – 112]	82 [56 – 134]	91 [50 – 135]
Fruit, grams/day	123 [65 – 232]	91 [40 – 166]	123 [65 – 231]	201 [98 – 261]
Potatoes, grams/day	119 [79 – 164]	119 [83 – 164]	123 [84 – 175]	119 [73 – 160]
Pasta, grams/day	19 [10–32]	19 [8–32]	18 [12–31]	26 [12–39]
Rice, grams/day	15 [4–30]	15 [4–25]	15 [0 – 25]	16 [4–32]
Legumes, grams/day	4 [0–11]	2 [0–11]	2 [0–11]	4 [0–17]
Nuts, grams/day	2 [0–4]	0 [0–2]	0 [0–3]	2 [0–6]
Beer, grams/day	0 [0–54]	0 [0–54]	0 [0–54]	0 [0–56]
Wine, grams/day	0 [0–20]	0 [0–0]	0 [0–10]	0 [20–106]
Fish, grams/day	11 [4–21]	10 [4–18]	10 [3–19]	17 [6–26]
Soy products, grams/day	0 [0–0]	0 [0–0]	0 [0–0]	0 [0–0]
Meat products, grams/day	94 [72–117]	95 [75 – 116]	91 [69 – 117]	93 [72–119]
Coffee, grams/day	500 [250–625]	375 [250 – 500]	500 [250 – 625]	500 [375–750]
Total plant protein intake, grams/day	29 [25–37]	27 [21–34]	29 [23–36]	32 [26–39]
Total animal protein intake, grams/day	48 [42–54]	49 [39–58]	49 [39–57]	55 [44–63]
Medication				
Prednisolone, *n* (%)	686 (99.0)	227 (98.3)	230 (99.6)	229 (99.1)
Calcineurin inhibitor, *n* (%)	397 (57.3)	135 (58.4)	133 (57.6)	129 (55.8)
Proliferation inhibitor, *n* (%)	573 (82.7)	189 (81.8)	193 (83.5)	191 (82.7)

*eGFR* estimated glomerular filtration rate as calculated using the creatinine and cystatin C-based CKD-EPI formula, *hs-CRP* high-sensitivity C-reactive protein

Tertile 1: boron excretion < 1040 µg/day, tertile 2: boron excretion between 1040 and 1540 µg/day, tertile 3: boron excretion > 1540 µg/day. Normally distributed data are presented as mean ± standard deviation, skewed data as median [interquartile range], and categorical data as number (valid percentage). Diabetes was defined according to the American Diabetes Association criteria [25]

Data on smoking status was missing in 46 patients (6.6%), data on donor age was missing in 19 patients (2.7%), data on eGFR was missing in 16 patients (2.3%), data on hs-CRP was missing in 43 patients (5.8%) and data on dietary intake was missing in 62 patients (8.9%). All other variables had missing data for < 10 patients

### Cross-sectional associations of 24 h urinary boron excretion

Univariable linear regression analyses showed that male sex and higher age were associated with higher urinary boron excretion (St. β = 0.10 and St. β = 0.08, respectively). Height, weight, and eGFR were positively associated with boron excretion, independent of age and sex (St. β = 0.23, St. β = 0.14 and St. β = 0.14, respectively, data not shown). Higher donor age and having a living kidney donor were associated with higher boron excretion, independent of age, sex, eGFR, height and weight. In contrast, boron excretion was independently, negatively associated with hs-CRP, leukocyte count, and homocysteine.

Wine, fruit and nut consumption were the strongest dietary determinants of boron excretion (St. β = 0.49, St. β = 0.26, and St. β = 0.25, respectively), independent of age, sex, eGFR, height and weight. Intake of fish and soy products was also independently associated with increased boron excretion, whereas intakes of potatoes and meat products were associated with lower boron excretion. In these analyses, it appeared that the positive association of age with boron intake disappeared if the association was adjusted for all the food groups that were significantly associated with urinary boron excretion (St. β for age = 0.01, 95% CI: − 0.04 to 0.06). Results of linear regression analyses of other clinical, dietary and biochemical parameters with urinary boron excretion are shown in Table 2.

**Table 2:** Univariable and multivariable linear regression analysis of log_2_ urinary boron excretion (µg/24 h)

	Univariable		Adj. for age, sex, eGFR, height and weight	
Baseline variables	St. β	95% CI	St. β	95% CI
Clinical characteristics				
Female sex	− 0.10	− 0.18 to − 0.03	0.06*	− 0.04 to 0.16
Age at visit	0.08	0.01 to 0.16	0.12*	0.04 to 0.19
Height	0.19	0.11 to 0.26	0.20*	0.09 to 0.31
Weight	0.16	0.09 to 0.24	0.09*	0.00 to 0.17
Body surface area	0.20	0.12 to 0.27	–	–
Body mass index	0.07	− 0.01 to 0.14	–	–
Systolic blood pressure	0.02	− 0.05 to 0.10	0.00	− 0.07 to 0.08
Diabetes	− 0.08	− 0.15 to 0.01	− 0.10	− 0.18 to − 0.02
History of CV disease	0.03	− 0.04 to 0.11	0.01	− 0.05 to 0.10
Current smoking	0.00	− 0.08 to 0.07	− 0.00	− 0.08 to 0.07
Pre-emptive transplantation	0.02	− 0.05 to 0.10	0.05	− 0.03 to 0.12
Time after transplantation^1^	− 0.02	− 0.09 to 0.06	− 0.01	− 0.08 to 0.07
Donor age	0.08	0.01 to 0.16	0.12	0.04 to 0.19
Living donor	0.14	0.07 to 0.21	0.15	0.07 to 0.23
Laboratory measurements				
Hemoglobin	0.18	0.11 to 0.25	0.11	0.02 to 0.19
Creatinine	− 0.11	− 0.18 to -0.04	–	
Cystatin C	− 0.20	− 0.27 to − 0.12	–	
eGFR	0.13	0.06 to 0.21	0.15*	0.08 to 0.23
Leukocyte count	− 0.15	0.22 to 0.07	− 0.13	− 0.20 to − 0.05
HDL cholesterol, mmol/L	0.10	0.02 to 0.17	0.13	0.06 to 0.21
LDL cholesterol, mmol/L	− 0.01	− 0.09 to 0.06	0.00	− 0.07 to 0.08
Triglycerides mmol/L	− 0.05	− 0.12 to 0.03	− 0.04	− 0.12 to 0.04
hs-CRP^1^	− 0.11	− 0.18 to -0.03	− 0.13	− 0.18 to − 0.02
24 h urinary protein excretion^1^	− 0.03	− 0.11 to 0.04	− 0.00	− 0.07 to 0.08
Homocysteine^1^	− 0.18	− 0.26 to -0.11	− 0.16	− 0.25 to − 0.07
Vitamin B12^1^	0.03	− 0.05 to 0.10	0.03	− 0.04 to 0.10
Vitamin B6^1^	0.19	0.12 to 0.27	0.20	0.13 to 0.27
Folic acid^1^	0.16	0.08 to 0.23	0.16	0.08 to 0.23
Dietary intake indexed by total energy intake				
Vegetables	0.02	− 0.05 to 0.10	0.04	− 0.04 to 0.12
Fruit ^2^	0.24	0.17 to 0.32	0.26	0.18 to 0.34
Potatoes	− 0.10	− 0.17 to − 0.02	− 0.13	− 0.22 to − 0.60
Pasta	− 0.02	− 0.10 to 0.06	0.01	− 0.07 to 0.09
Rice	0.04	− 0.04 to 0.12	0.06	− 0.02 to 0.14
Legumes^2^	0.05	− 0.03 to 0.13	0.04	− 0.04 to 0.12
Nuts^2^	0.23	0.15 to 0.31	0.25	0.18 to 0.33
Beer^2^	0.04	− 0.04 to 0.12	0.00	− 0.08 to 0.09
Wine^2^	0.48	0.42 to 0.55	0.49	0.42 to 0.56
Fish^2^	0.11	0.04 to 0.19	0.12	0.04 to 0.19
Soy products^2^	0.08	0.01 to 0.16	0.11	0.03 to 0.18
Meat products	− 0.12	− 0.20 to − 0.05	− 0.12	− 0.20 to − 0.05
Coffee	0.10	0.02 to 0.18	0.06	− 0.02 to 0.13
Total plant protein intake	0.12	0.04 to 0.20	0.11	0.04 to 0.19
Total animal protein intake	0.01	− 0.07 to 0.09	0.01	− 0.07 to 0.09
Medication use				
Prednisolone	0.03	− 0.05 to 0.10	0.03	− 0.04 to 0.10
Calcineurin inhibitor	− 0.08	− 0.15 to − 0.00	− 0.05	− 0.13 to 0.02
Proliferation inhibitor	0.04	− 0.04 to 0.11	0.02	− 0.06 to 0.09

*eGFR* estimated glomerular filtration rate as calculated using creatinine and cystatin C-based CKD-EPI formula, *hs-CRP* high-sensitivity C-reactive protein

*Regression coefficients of models which only contain age, sex, eGFR, height, and weight as independent variables. Associations of body surface area and body mass index were not multivariable adjusted because of collinearity with body weight and associations of creatinine and cystatin C were not multivariable adjusted because of collinearity with eGFR. Smoking status was missing in 46 patients (6.6%), eGFR was missing in 16 patients (2.3%) and donor age in 19 patients (2.7%), hs-CRP was missing in 43 patients (5.8%), vitamin B6 was missing in 11 patients (1.6%) and data on dietary intake was missing in 62 patients (8.9%). Other variables had missing data for < 10 patients

^1^Variables were log_2_ transformed

^2^Variables were transformed using a square root. Diabetes was defined according to the American Diabetes Association criteria [25]

### Longitudinal associations of 24 h urinary boron excretion with all-cause mortality

**Fig. 1:**
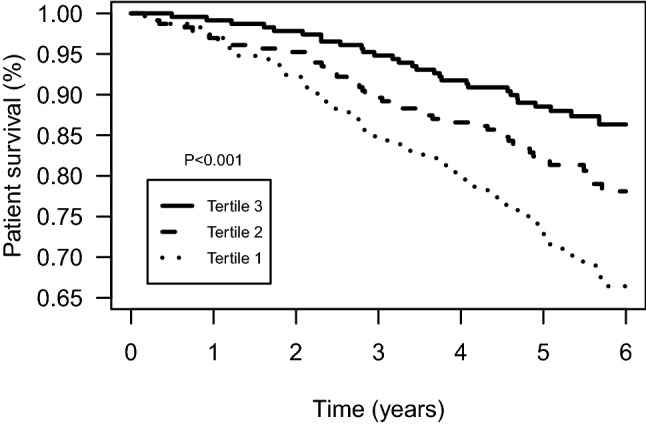
Kaplan–Meier plot for all-cause mortality per tertile of 24 h urinary boron excretion. Tertile 1: < 1040 µg/24 h. Tertile 2: 1040–1540 µg/24 h. Tertile 3: > 1540 µg/24 h. *P* value represents the evidence against the null hypothesis of no difference in survival across tertiles, as calculated using log-rank test.

During median follow-up of 5.4 [4.8 to 6.1] years, 150 (22%) patients died. Mortality rates among the lowest, middle, and highest tertiles of boron were 73 (32%), 47 (20%), and 30 (13%), respectively (*P*_log-rank_ < 0.001, Fig. 1). Patients in tertiles 2 and 3 of boron excretion had lower risk of mortality (HR: 0.59, 95% CI: 0.41 to 0.86, and HR: 0.36, 95% CI: 0.24 to 0.56, respectively) compared with patients in tertile 1 (Table 3). The association of tertile 3 of 24 h boron excretion with all-cause mortality remained, independent of potential confounders, including age, sex, eGFR, history of cardiovascular disease, smoking status, diabetes mellitus, height, weight, LDL cholesterol, HDL cholesterol, and triglycerides (HR: 0.43, 95% CI: 0.27 to 0.67), whereas the association of tertile 2 with mortality was no longer statistically significant (HR: 0.73, 95% CI: 0.50 to 1.08). The associations of tertile 3 of urinary boron excretion with lower risk of mortality also remained after further adjustment for the energy-indexed intake of fruit, nuts, fish, wine, plant protein and animal protein.

**Table 3:** Cox regression analysis of the 24 h urinary boron excretion with all-cause mortality. Presented models are cumulative, and add variables to the model in each step

Model	Tertiles of 24 h urinary boron excretion					Continuous analyses of 24 h urinary boron excretion	
	1	2 1040 to 1540 µg/day		3 > 1540 µg/day		Per doubling	
		HR (95%CI)	*P* value	HR (95% CI)	*P* value	HR (95% CI)	*P* value
Univariable	Ref	0.59 (0.41–0.86)	0.006	0.36 (0.24–0.56)	< 0.001	0.55 (0.44–0.70)	< 0.001
+ age, sex, eGFR, CV history	Ref	0.70 (0.48–1.03)	0.068	0.35 (0.23–0.54)	< 0.001	0.51 (0.40–0.66)	< 0.001
+ smoking status, diabetes	Ref	0.70 (0.48–1.03)	0.068	0.37 (0.24–0.58)	< 0.001	0.53 (0.41–0.69)	< 0.001
+ height and weight	Ref	0.72 (0.49–1.06)	0.093	0.41 (0.26–0.63)	< 0.001	0.56 (0.43–0.73)	< 0.001
+ LDL cholesterol, HDL cholesterol, triglycerides	Ref	0.73 (0.50–1.08)	0.1	0.43 (0.27–0.67)	< 0.001	0.58 (0.48–0.76)	< 0.001
+ dietary intake*	Ref	0.77 (0.52–1.14)	0.2	0.51 (0.30–0.84)	0.009	0.63 (0.46–0.86)	0.004

*CI* confidence interval, *eGFR* estimated glomerular filtration rate as calculated using creatinine and cystatin C-based CKD-EPI equation, *HR* hazard ratio, *SD* standard deviation

*In this model, we adjusted for age, sex, eGFR, CV history, smoking status, diabetes, LDL cholesterol, HDL cholesterol, triglycerides, and energy intake-indexed intake of fruit, nuts, fish, wine, plant protein and animal protein. Parameters of dietary intake were transformed using a square root. 150 patients (22%) died during a median follow-up time of 5.4y [4.8–6.1y]. Addition of log_2_ 24 h urinary boron excretion significantly augmented the model of sex, age, eGFR and history of cardiovascular disease (*P*_likelihood ratio_ < 0.001). Multiple imputations was used to account for missing data in 16 cases (2.3%) for eGFR, 46 cases (6.6%) for smoking status, and 62 patients (8.9%) for dietary intake. Other variables had missing data for < 10 patients

**Fig. 2:**
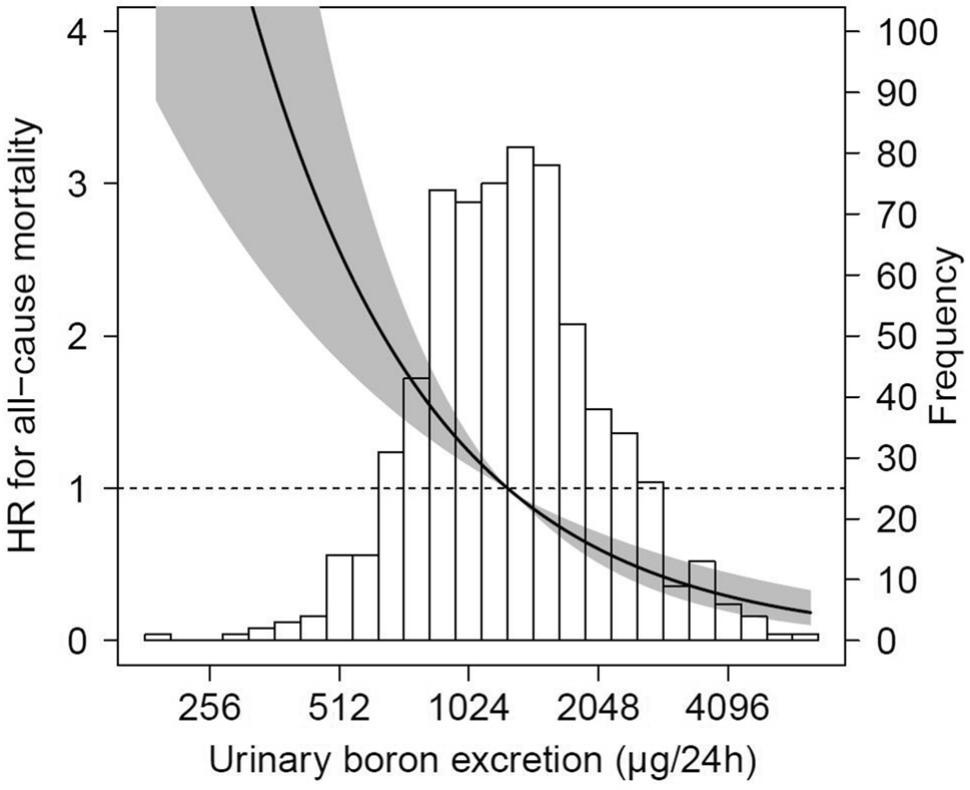
Graphical representation of the association between 24 h urinary boron excretion and risk of all-cause mortality, based on a Cox proportional hazards regression analyses with median boron excretion (1275 µg/24 h) as reference. The model was adjusted for age, sex, eGFR, and history of cardiovascular disease, and presented in relation to the histogram of urinary boron excretion. The black line represents the hazard ratio, the grey area represents the 95% confidence interval. *HR* hazard ratio

Cox proportional hazards analyses with 24 h urinary boron excretion as a continuous variable also showed strong associations with mortality, independent of adjustment for age, sex, eGFR, history of cardiovascular disease, as visualized in Fig. 2. Additional adjustment for smoking status, diabetes mellitus, height, weight, LDL cholesterol, HDL cholesterol, and triglycerides did not materially change this association (HR per doubling of boron excretion: 0.58, 95% CI: 0.48 to 0.76, Table 3). Again, these associations remained fundamentally unchanged after additional adjustment for the energy-indexed intake of mentioned food groups (HR: 0.63, 95% CI: 0.46 to 0.86). We found no interactions of boron excretion with age, sex, and eGFR for the association with mortality.

### Secondary analyses with graft failure

During a median follow-up of 5.4 [4.8 to 6.1] years, a total of 83 (12%) patients experienced graft failure. Boron excretion on the continuous scale was associated with death-censored graft failure in the crude model (HR: 0.63, 95% CI: 0.46 to 0.87), but this association was not statistically significant after adjustment for age, sex, eGFR, and history of cardiovascular disease (HR: 0.89, 95% CI: 0.62 to 1.27).

### Sensitivity analyses

In sensitivity analyses, we performed the Cox proportional hazards analyses including the predefined variables potentially in the causal path between boron and mortality (Table 4). The associations of tertile 3 of boron excretion with lower risk of mortality remained materially unchanged in the full model (HR: 0.42, 95% CI: 0.27 to 0.66). The association between boron excretion on a continuous scale and mortality also remained materially unchanged (HR per doubling of boron excretion: 0.59, 95% CI: 0.46 to 0.77).

**Table 4:** Causal path analyses of the association of boron excretion with all-cause mortality. Presented models are cumulative, and add variables to the model in each step

Models	Tertiles of 24 h urinary boron excretion					Continuous analyses of 24 h urinary boron excretion	
	1	2 1040 to 1540 µg/day		3 > 1540 µg/day		Per doubling	
		HR (95%CI)	*P* value	HR (95% CI)	*P* value	HR (95% CI)	*P* value
Age, sex, eGFR, CV history, smoking, diabetes, height, weight	Ref	0.72 (0.49–1.06)	0.093	0.41 (0.26–0.63)	< 0.001	0.56 (0.43–0.73)	< 0.001
+ hs-CRP^a^	Ref	0.73 (0.50–1.08)	0.1	0.41 (0.26–0.65)	< 0.001	0.57 (0.44–0.75)	< 0.001
+ homocysteine^a^	Ref	0.73 (0.50–1.08)	0.1	0.42 (0.27–0.66)	< 0.001	0.58 (0.44–0.75)	< 0.001
+ vitamin B6, B12, folic acid^a^	Ref	0.76 (0.52–1.13)	0.2	0.42 (0.27–0.66)	< 0.001	0.59 (0.46–0.77)	< 0.001

*CI* confidence interval, *eGFR* estimated glomerular filtration rate as calculated using creatinine and cystatin C-based CKD-EPI equation, *HR* hazard ratio, *SD* standard deviation

150 patients (22%) died during a median follow-up time of 5.4y [4.8–6.1y]. Multiple imputations was used to account for 16 missing values (2.3%) of eGFR, 46 (6.6%) missing values for smoking status, 8 missing values (1.1%) for vitamin B12 and folic acid, 11 missing values (1.6%) for vitamin B6, and 43 missing values (5.8%) of hs-CRP

^a^Variables were log_2_ transformed

In further sensitivity analyses, the associations of urinary boron excretion with mortality remained materially unchanged in Cox proportional hazards analyses after exclusion of the 81 KTR reporting current smoking, as presented in Supplementary Table 2. The strong association of boron excretion with lower risk of mortality remained, independent of adjustment for age, sex, eGFR, CV history, smoking status, diabetes, height, weight, LDL and HDL cholesterol, triglycerides, and dietary intake; HR: 0.61, 95% CI: 0.43 to 0.87). The analyses with adjustment for parameters potentially in the causal path also remained materially unchanged (HR: 0.56, 95% CI: 0.41 to 0.76, Supplementary Table 3).

## Discussion

This study is the first to assess cross-sectional and prospective associations of boron excretion in KTR. Wine, fruit and nut consumption were strongly associated with increased boron excretion. In contrast, homocysteine, hs-CRP and leukocyte count were negatively associated with boron excretion. Prospective analyses demonstrated that a higher 24 h urinary boron excretion, reflecting higher boron exposure [22], was independently associated with a lower risk of mortality in KTR.

Currently, no generally accepted reference values for urinary boron excretion exist. However, the observed 24 h urinary boron excretion rates in our study are consistent with previous studies in Japan and the UK [30, 31]. In contrast, a population of 119 healthy subjects in Italy had higher boron excretions [32]. This difference may be explained by geographical differences, as boron concentrations are reportedly high near the Mediterranean Sea [3]. In addition, the higher boron excretion rates in the Italian study may be explained by diet, since Mediterranean diets generally include a higher intake of plant-derived foods, such as boron-rich fruit, wine and nuts, which are the current main sources of human boron ingestion [5, 6, 33]. Indeed, our results confirm the strong association of fruit and wine intake with urinary boron excretion. Dietary factors also appeared to explain the observed positive association of age with boron excretion. Other studies have also indicated that 24 h urinary boron excretion is a useful reflection of recent boron intake. For example, a study among 18 healthy volunteers concluded that the absorption efficiency of dietary boron intake is high [34]. Another study by Hunt and colleagues, showed that a 9.0-fold increase in oral boron intake showed a modest 1.5-fold in circulating plasma concentrations, but no signs of boron accumulation over time [35]. Both studies report that > 80% of oral boron intake was retrieved in urine, and both conclude that urinary boron excretion may provide a useful reflection of boron intake. More recently, a study has been published on the effects of the intake of mineral waters with either low, medium or high boron content on 24 h urinary boron excretion in humans [23]. Notably, 24 h urinary boron excretion strongly increased after intake of mineral water with high boron content (3262 ± 430 µg/24 h in the high boron group, compared to 1081 ± 386 µg/24 h in the low boron group, respectively). The observed associations of dietary intake with boron excretion in our study further support that 24 h urinary boron excretion reflects dietary boron intake.

The most important finding of this study is the strong independent association of 24 h urinary boron excretion with lower risk of mortality, but not graft failure. The association between higher boron excretion and lower risk of mortality may seem counterintuitive since boron used to have a reputation of being potentially harmful after several reports in the twentieth century. For example, several cases of infants with boron intoxication as a result of dipping tranquilizers in boric acid have been reported [36], and a study into the effects of preservatives indicated that ingestion of (the equivalence of) 4 to 5 g of borax per day may result in “loss of appetite and inability to perform work of any kind” [37]. However, the boron exposure levels in our study are two orders of magnitude lower than those described in the mentioned reports. Boron intake of up to 0.4 mg/kg body weight (32 mg/day for a 80 kg person, i.e. more than five times higher than the highest measurement in the current study) is considered safe by the WHO [14, 38].

The found associations of boron with lower risk of mortality are in line with geographical risk assessment studies, suggesting a decreased risk of cancer and mortality in populations with more boron exposure [17, 18]. For example, Barranco and colleagues showed that increased boron concentrations in groundwater correlated with reduced risk of prostate cancer and mortality, in a study performed in Texas, USA [17]. In a geographical risk assessment study in Northern France, Yazbeck and colleagues reported that there is a tendency toward lower mortality rates in municipalities with high boron content in drinking water [18]. Additionally, high boron exposure is an underappreciated common factor in all of the Blue Zones, further suggesting beneficial effects of boron on health and longevity. Studies in *Drosophila*, that showed an increased life span of 9.5% in flies receiving low-dose boron supplementation, provide further support for the potential beneficial effects of boron on longevity [15]. The current study appears to further indicate potential beneficial effects of boron in humans, especially because the associations between higher boron excretion and decreased risk of mortality remained statistically and clinically significant, even after adjustment for multiple confounders.

Based on animal and human studies, multiple effects of boron may explain the association between higher boron excretion and lower risk of mortality. First, boron appears to play a role in the homocysteine-methionine pathway, which is involved in many biochemical reactions within the human body, including methylation of DNA, neurotransmitters, and many proteins [21, 39]. For example, a study in 2009 showed that boron deprivation increased homocysteine and decreased *S*-adenosylmethionine concentrations in rats, indicating that boron may exert part of its beneficial effects through the homocysteine-methionine pathway [21]. One may hypothesize that these findings, together with the notion that increased homocysteine and decreased *S*-adenosylmethionine concentrations are associated with many human diseases [21], may in part explain the association between boron excretion and lower risk of mortality. Although our current study cannot provide evidence for causality, the negative association between boron excretion and homocysteine further suggests the involvement of boron in the homocysteine-methionine pathway. However, the association between boron excretion and mortality was not affected by additional adjustment for homocysteine concentrations, which renders it unlikely that homocysteine is the major mediator in this association. The association between boron excretion and mortality was also not materially affected by adjustment for CRP and leukocyte count and by adjustment for blood lipids, also rendering it unlikely that inflammation or blood lipids were main mediators in this association.

These results suggest that boron may exert its potential beneficial effects on survival, through pathways other than the homocysteine–methionine cycle, inflammation or blood lipids. Another proposed mechanism for the beneficial effects of boron on longevity is through its binding to NAD^+^ and cADPR, by which boron may counteract oxidative stress, and influence DNA repair, telomere stability, and ageing process [40]. Future studies are warranted to further clarify the physiological functions of boron, and assess whether this putative mechanism may indeed explain our findings. Although underlying mechanisms are only partly elucidated, the beneficial effects of boron have become increasingly clear in the past decades [14, 40]. Since many people consistently consume less than 1 mg boron per day, which has been suggested by the WHO as the minimal safe mean population dietary intake, boron deficiency may be a major overlooked nutritional problem worldwide [8, 33, 38]. Moreover, our study suggests beneficial effects of increasing boron intake to levels well over 1 mg per day.

Strengths of our study are the long follow-up and the large study population. In addition, extensive data collection of many demographic, dietary and laboratory parameters enabled adjustment for many potential confounders. The primary analyses of the study concerned the association of urinary boron excretion with all-cause mortality, which is a single hypothesis to be tested, where multivariable models are applied to test robustness of the association to adjustment for potential confounders. Formally, this does not require correction for multiple testing, and we, therefore, did not correct for multiple testing in this manuscript. However, it should be mentioned that our findings concerning the primary analyses were highly significant, so that the results remained materially unchanged even if corrections for multiple testing were applied for each adjustment model. However, several limitations of this study need to be addressed. For instance, the current study was performed in the northern part of The Netherlands, which is home to a predominantly Caucasian population consuming a predominantly Dutch diet, which calls prudence to extrapolation of our results to other populations. This study should therefore be replicated in other populations, from different geographical areas, consuming different diets. To the best of our knowledge, it is unknown whether tubular boron reabsorption varies with differences in urinary boron excretion. Assessment of tubular boron reabsorption would require data on plasma boron concentrations in addition to urinary boron excretion. Unfortunately, data on circulating boron concentrations were not available in our study. Future studies may study boron concentrations in blood, and assess tubular reabsorption rate and fractional excretion rate of boron to further clarify the underlying mechanisms of our findings. Additionally, food composition tables generally do not include information on boron content, which prevented us from comparing FFQ-based boron intake to 24 h urinary excretion of boron. Although several studies suggest that 24 h urinary boron excretion reflects intake, it remains unknown what time frame of exposure a 24 h urine collection might reflect, and studies that evaluated 24 h urinary boron excretion as a reliable and valid biomarker of intake are lacking. If our finding of an association of 24 h urinary boron excretion with long-term outcome would be replicated in other longitudinal cohort studies, and intervention studies would be designed, it would become increasingly relevant to generate data on reliability and validity of 24 h urinary boron excretion as a biomarker of intake. Another limitation is that our study population consists of patients who survived at least one year after kidney transplantation. It cannot be excluded that this introduced collider bias, which might limit extrapolation of our findings to other populations. In addition, due to its observational design, this study can only describe associations and cannot prove causality. Despite adjustment for self-reported dietary intake and circulating vitamin concentrations in blood, it is conceivable that boron excretion might be a marker of overall diet quality or consumption of plant-based foods, rather than an actual causal agent. Only randomized controlled trials can assess the potential causal effects of boron exposure on health and disease.

In conclusion, in this single-center cohort of kidney transplant recipients, we showed that high 24 h urinary boron excretion is associated with a lower risk of premature death, independent of potential confounders. Since 24 h urinary boron excretion can provide a useful reflection of daily boron exposure, these results suggest that increased boron intake may potentially decrease the risk of premature mortality in these patients. Randomized trials with dietary interventions are needed to assess the potential of increased boron intake in kidney transplant recipients.

## Supplementary Information

Below is the link to the electronic supplementary material.

Supplementary file1 (DOCX 128 KB)

## Data Availability

Data described in the manuscript, code book, and analytic code will be made available upon request.

## Abbreviations

95% CI: 95% Confidence interval
eGFR: Estimated glomerular filtration rate
HR: Hazard ratio
hs-CRP: High-sensitivity C-reactive protein
ICP-MS: Inductively coupled plasma mass spectrometry
IQR: Interquartile range
KTR: Kidney transplant recipients
