# Supplementary material for: Boron Intake and decreased risk of mortality in kidney transplant recipients
Source: Eur J Nutr. 2021 Oct 22;61(2):973–84. doi: 10.1007/s00394-021-02702-0 (PMC8854244; doi:10.1007/s00394-021-02702-0)
Supplement: Supplementary file 1 — Supplementary file1 (DOCX 128 KB) [file 394_2021_2702_MOESM1_ESM.docx]

**Supplementary files**

**Supplementary Table 1 |** Boron analysis using inductively coupled plasma mass spectrometry (ICP-MS).

| **Experimental conditions** |  |
| --- | --- |
| **Apparatus** | ICAPQ (Thermo Fisher Scientific Waltham) |
| **Method** | DIN EN ISO 17294-2: 2017-01  Conducted by SYNLAB Analytics & Service,  Jena, Germany |
| **Final sample dilution** | 1 to 50 (2 % (v/v)) nitric acid |
| **Internal standard** | Rhodium (2 µg/L) |
| **Limit of detection (LOD)** | < 1.3 µg/L |
| **Limit of quantification (LOQ)** | 5 µg/L |
| **Recovery** | 101.8 % (n=6) |
| **Intra-day precision, coefficient of variation** | 2.35 % (n=6) |

**Supplementary Table 2 |** Cox regression analysis of the 24h urinary boron excretion with all-cause mortality after exclusion of patients reporting current smoking (N = 612, 88.3% of total study population).

|  | **Tertiles of 24h urinary boron excretion^#^** | | | | | **Continuous analyses of  24h urinary boron excretion** | |
| --- | --- | --- | --- | --- | --- | --- | --- |
|  | **1** | **2**  **1040 to 1540 µg/day** | | **3**  **>1540 µg/day** | | **Per doubling** | |
| **Model** |  | **HR (95%CI)** | **P-value** | **HR (95% CI)** | **P-value** | **HR (95% CI)** | **P-value** |
| Univariable | *Ref.* | 0.62 (0.42 – 0.91) | 0.016 | 0.38 (0.24 – 0.60) | <0.001 | 0.55 (0.42 – 0.71) | <0.001 |
| + age, sex, eGFR, CV history | *Ref.* | 0.75 (0.50 – 1.13) | 0.2 | 0.38 (0.24 – 0.61) | <0.001 | 0.53 (0.40 – 0.69) | <0.001 |
| + diabetes | *Ref.* | 0.66 (0.42 – 1.02) | 0.063 | 0.39 (0.24 – 0.64) | <0.001 | 0.52 (0.38 – 0.70) | <0.001 |
| + height, weight | *Ref.* | 0.67 (0.43 – 1.05) | 0.080 | 0.42 (0.25 – 0.69) | <0.001 | 0.53 (0.39 – 0.72) | <0.001 |
| + LDL cholesterol, HDL cholesterol, triglycerides | *Ref.* | 0.70 (0.44 – 1.09) | 0.1 | 0.44 (0.27 – 0.73) | 0.002 | 0.55 (0.41 – 0.75) | <0.001 |
| + dietary intake* | *Ref.* | 0.76 (0.48 – 1.19) | 0.2 | 0.54 (0.31 – 0.94) | 0.030 | 0.61 (0.43 – 0.87) | 0.007 |

^#^ For consistency, tertiles were defined based upon the entire study population, prior to exclusion of current smokers. *In this model, we adjusted for age, sex, eGFR, CV history, diabetes, and energy intake-indexed fruit, nuts, fish, wine, plant protein and animal protein intake. Parameters of dietary intake were transformed using a square root. 117 patients (19%) died during a median follow-up time of 5.4y [4.9 – 6.1y]. Addition of log_2_ 24h urinary boron excretion significantly augmented the model of sex, age, eGFR and history of cardiovascular disease (P_likelihood ratio_ < 0.001). Multiple imputation was used to account for 15 missing values (2.5%) of eGFR, 9 missing values (1.5%) of LDL and HDL cholesterol, 8 missing values (1.3%) of triglycerides, and 55 missing values (9.1%) for dietary intake parameters. CI, confidence interval; eGFR, estimated glomerular filtration rate as calculated using creatinine and cystatin C-based CKD-EPI equation; HR, hazard ratio; SD, standard deviation.

**Supplementary Table 3 |** Causal path analyses of the association of boron excretion with all-cause mortality after exclusion of patients reporting current smoking (N = 612, 88.3% of total study population). Defined models are cumulative, and add variables to the model in each step.

|  | **Tertiles of 24h urinary boron excretion^#^** | | | | | **Continuous analyses of  24h urinary boron excretion** | |
| --- | --- | --- | --- | --- | --- | --- | --- |
|  | **1** | **2**  **1040 to 1540 µg/day** | | **3**  **>1540 µg/day** | | **Per doubling** | |
| **Models** |  | **HR (95%CI)** | **P-value** | **HR (95% CI)** | **P-value** | **HR (95% CI)** | **P-value** |
| Age, sex, eGFR, CV history, diabetes, height, weight | *Ref.* | 0.67 (0.43 – 1.05) | 0.080 | 0.42 (0.25 – 0.69) | <0.001 | 0.53 (0.39 – 0.72) | <0.001 |
| + hs-CRP ^a^ | *Ref.* | 0.69 (0.44 – 1.08) | 0.1 | 0.42 (0.26 – 0.70) | <0.001 | 0.54 (0.40 – 0.73) | <0.001 |
| + homocysteine ^a^ | *Ref.* | 0.70 (0.45 – 1.09) | 0.1 | 0.43 (0.26 – 0.72) | 0.001 | 0.55 (0.40 – 0.75) | <0.001 |
| + vitamin B6, B12, folic acid ^a^ | *Ref.* | 0.70 (0.45 – 1.10) | 0.1 | 0.44 (0.27 – 0.73) | 0.002 | 0.56 (0.41 – 0.76) | <0.001 |

^#^ For consistency, tertiles were defined based upon the entire study population, prior to exclusion of current smokers. 117 patients (19%) died during a median follow-up time of 5.4y [4.9 – 6.1y]. Multiple imputation was used to account for 15 missing values (2.5%) of eGFR, 7 missing values (1.2%) for vitamin B12 and folic acid, 11 missing values (1.8%) for vitamin B6 and 35 missing values (5.8%) of hs-CRP. ^a^variables were log_2_ transformed. CI, confidence interval; eGFR, estimated glomerular filtration rate as calculated using creatinine and cystatin C-based CKD-EPI equation; HR, hazard ratio; SD, standard deviation.


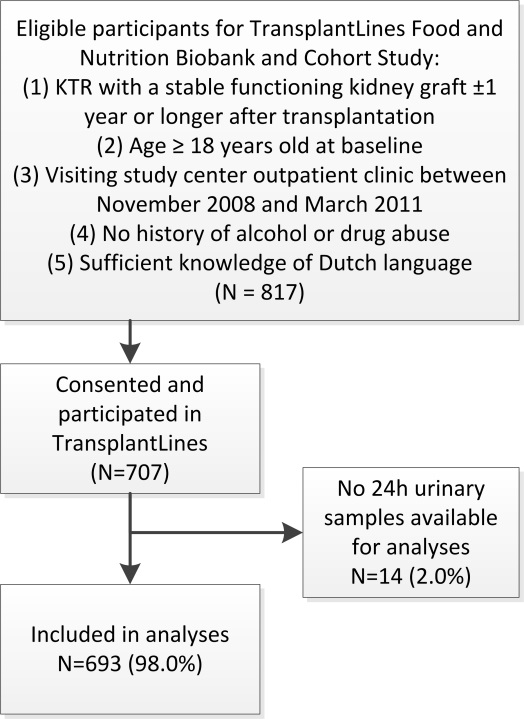


**Supplementary Figure 1 |** Flow of participants through the study.
